# OX40 agonist immunotherapy expands tumor reactive CD8 T cells with a unique T cell receptor repertoire and synergizes with PDL-1 blockade to promote tumor regression

**DOI:** 10.1186/2051-1426-2-S3-O18

**Published:** 2014-11-06

**Authors:** Amy Moran, Fanny Polesso, Andrew Weinberg

**Affiliations:** 1 Earle A Chiles Cancer Research Institute/Providence Cancer Center, Portland, OR, USA

## 

Previously, the field of tumor immunology has relied on T cell receptor (TCR) transgenic mice and model tumor antigens to study the T cell:tumor cell interaction. However, these models have limitations. Namely, they do not reflect the natural affinity, repertoire, or precursor frequency of tumor antigen-specific T cells. Using a novel TCR affinity tool, the Nur77GFP mouse, we have characterized the signal strength and phenotype of naturally occurring tumor antigen-specific CD8 T cells. Our data suggest that the co-expression of Nur77GFP and PD-1 identifies a population of polyclonal CD8+ tumor infiltrating lymphocytes (TIL) that are tumor antigen-specific. This population produces more IFN-γ *in situ *and upon re-stimulation with syngeneic but not allogeneic tumor cells. In addition, treatment of tumor bearing mice with an OX40 agonist mAb doubled the frequency of tumor reactive T cells receiving strong TCR signals as compared to the control (~two-fold increase in CD8+Nur77GFPhi p=.0027). PD-1, PDL-1, or CTLA4 blockade also had a modest influence on the frequency of CD8+Nur77GFPhi TIL but this was not as significant as anti-OX40 mAb. To further understand the anti-tumor immune response, CD8+Nur77GFPhi and Nur77GFPlo TIL were sorted and the TCRb CDR3 region was sequenced. We observed clonal expansions of unique Vb sequences exclusively found in the TIL population after anti-OX40 immunotherapy. Unexpectedly, the CD8+ Nur77GFPlo population sorted from mice treated with the anti-OX40 mAb contained very similar Vb sequences and together these represented more than 30% of the TCR repertoire. This suggests that both weak and strong tumor antigens drive TIL expansion after anti-OX40 mAb immunotherapy. Importantly, these repertoire changes were not observed with the control antibody in TIL nor in the spleens of OX40 agonist treated mice. OX40 agonist therapy also increased the frequency of PD-1+ TIL suggesting that targeting this inhibitory molecule in combination with OX40 might improve tumor rejection. In fact, when established tumors (~50mm^2^) were treated with a combination of anti-OX40 and anti-PDL-1, significant tumor regression and disease free survival was observed in multiple tumor models (C57Bl/6:MCA205 50% survival p < 0.0001, Balb/c:CT26 30% survival p = 0.0105). Tumor rejection corresponded to an increase in the circulation of CD44hi, proliferating, CD8 T cells that was observed only in the combination therapy group as compared to either single agent alone. Therefore, we propose that one mechanism of OX40 agonist immunotherapy is via the priming and expansion of low and high affinity tumor antigen specific CD8 T cells.

